# Effective Solid Electrolyte Interphase Formation on Lithium Metal Anodes by Mechanochemical Modification

**DOI:** 10.1021/acsami.1c07490

**Published:** 2021-07-15

**Authors:** Julia Wellmann, Jan-Paul Brinkmann, Björn Wankmiller, Kerstin Neuhaus, Uta Rodehorst, Michael R. Hansen, Martin Winter, Elie Paillard

**Affiliations:** † Forschungszentrum Jülich GmbH (IEK-12) Helmholtz-Institute Münster, Corrensstraße 46, Münster 48149, Germany; ‡ Institute of Physical Chemistry, University of Münster, Corrensstraße 28-30, Münster 48149, Germany; § MEET Battery Research Center, University of Münster, Corrensstraße 46, Münster 48149, Germany; ◊ Department of Energy, Politecnico di Milano, Via Lambruschini 4, Milan 20156, Italy

**Keywords:** ionic liquids, lithium metal anodes, lithium metal batteries, mechanochemical modification, solid electrolyte interphase

## Abstract

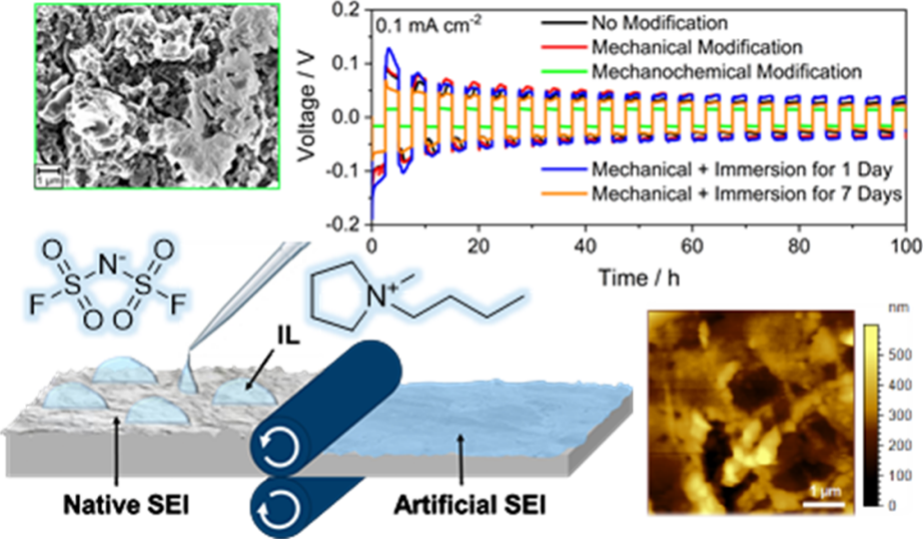

Lithium metal batteries are gaining increasing attention due to their potential for significantly higher theoretical energy density than conventional lithium ion batteries. Here, we present a novel mechanochemical modification method for lithium metal anodes, involving roll-pressing the lithium metal foil in contact with ionic liquid-based solutions, enabling the formation of an artificial solid electrolyte interphase with favorable properties such as an improved lithium ion transport and, most importantly, the suppression of dendrite growth, allowing homogeneous electrodeposition/-dissolution using conventional and highly conductive room temperature alkyl carbonate-based electrolytes. As a result, stable cycling in symmetrical Li∥Li cells is achieved even at a high current density of 10 mA cm^–2^. Furthermore, the rate capability and the capacity retention in NMC∥Li cells are significantly improved.

## 1 Introduction

Nowadays, established energy sources such as fossil fuels and nuclear power plants are getting replaced by more environmentally friendly and renewable energy sources, *e.g.*, wind and solar power. Due to the discontinuity in power delivery, there is a demand for reliable energy storage systems to offset these fluctuations. Additionally, interest in electric vehicles is strongly increasing. While lithium ion batteries, using a layered metal oxide cathode, an alkyl carbonate-based liquid electrolyte, and a graphite-based anode, are the state-of-the-art technology for many applications and still have a bright future, alternative electrode materials with even higher specific capacities are being investigated for having more technology options in future energy storage.^1−6^

Lithium metal is a promising anode candidate since it has the most negative standard reduction potential of all metals (−3.04 V *vs* standard hydrogen electrode (SHE)) and a high theoretical specific capacity of 3861 mAh g^–1^. However, it suffers from several drawbacks that must be addressed to allow a broad application of lithium metal batteries (LMBs).^7^ Lithium metal anodes react with the electrolyte, forming the so-called solid electrolyte interphase (SEI) that typically consists of a dense inorganic inner layer made of LiF, Li_2_O, Li_2_CO_3_, and other inorganic salts covered by a more porous and organic outer layer consisting of organic molecular and ionic compounds. The SEI is usually inhomogeneous in terms of thickness and lithium ion conductivity, which favors electrodeposition/-dissolution of lithium at/through the more conductive and thinner parts of the SEI. This causes high-surface-area lithium (HSAL) formation with various morphologies, *e.g.*, dendritic and/or mossy, depending on the used electrolytes and operation conditions. On the one hand, this lowers the Coulomb efficiency (CE) and cell specific capacity due to the formation of electronically disconnected “dead” lithium and degradation products, which may, over cycling, form a layer that hinders lithium ion transport. On the other hand, HSAL can grow through the separator/liquid electrolyte, leading to short circuits, raising the risk of thermal runaway and therefore posing serious safety issues.^8−12^

In order to diminish or even prevent unwanted reactions between the electrodes and the electrolyte, the anode and the cathode potentials have to be within the electrochemical stability window of all electrolyte components, which, in theory, means that the anode potential has to be lower than the lowest unoccupied molecular orbital of the electrolyte and the cathode potential has to be higher than the highest occupied molecular orbital of the electrolyte. However, in real electrolytes, there are differences to theory, *e.g.*, due to interactions between various compounds that can change the energy levels of the orbitals and the existence of decomposition pathways involving several electrolyte components. Therefore, in addition to this thermodynamic limitation, usually a kinetic barrier (*i.e.*, a protective layer on the electrode) is needed.^13−15^ In the case of lithium metal, an effective SEI is required, especially when cycling with organic solvent-based electrolytes is intended. An ideal SEI offers a high lithium ion conductivity while blocking other ionic species as well as electrolyte solvents, and it should be electronically insulating. Furthermore, it should not react with the electrolyte and be homogeneous in terms of lithium ion conductivity and thickness. Mechanical strength and, at the same time, some flexibility are also required to prevent dendrite penetration and to buffer the volume change during cycling.^16^

Several approaches have been proposed to form effective SEIs and to improve the safety and performance of lithium metal anodes. Many studies focus on new electrolyte formulations including SEI forming additives, solid polymer electrolytes, or highly concentrated electrolytes.^17,18^ Unfortunately, electrolyte additives usually get consumed during cycling and can therefore only improve short-term cycling.^19−21^ In highly concentrated electrolytes, the majority of solvent molecules coordinate to salt cations, which suppresses reactions between lithium metal and free solvent molecules. Nevertheless, they are expensive and viscous due to the high salt content.^22−24^ Solid polymer electrolytes significantly enhance the safety of the cells and allow a good wetting and mechanical confinement of lithium metal, especially at higher temperatures where lithium metal is more ductile. However, they usually suffer from very low ionic conductivity at ambient temperature.^25^ In search of a good compromise between lithium ion conductivity and safety, ionic liquids (ILs), which are room temperature molten salts consisting of large organic cations and anions with well-delocalized negative charges, receive continuous attention. However, although they possess excellent SEI forming properties and compatibility with lithium metal, their ionic conductivities are still considerably lower than those of conventional organic solvent-based electrolytes while their cost is considerably higher.^26−28^ Finally, even though the presence of extra ions acting as a supporting electrolyte is favorable to act as an electrostatic shield that could limit the extension of electrical field gradients into the electrolyte (and thereby limiting HSAL nucleation), they also result in low lithium transference numbers in the bulk electrolyte and thus lead to strong ion concentration gradients causing lithium ion depletion and thereby may favor diffusion-controlled dendrite growth.^29,30^

Apart from electrolyte formulations, surface treatments can be applied prior to cell assembly. For instance, mechanical methods such as roll-pressing or micropatterning and chemical modifications by physical vapor deposition or immersion have been reported.^31−35^ Roll-pressing “dilutes” the “native” film (*i.e.*, the layer formed during lithium metal production and processing prior to contact with the electrolyte), which mainly consists of Li_2_O and Li_2_CO_3_, and smoothens the lithium metal, resulting in a more homogeneous surface.^31^ However, due to the thinner “native” film (thus a “cleaner” surface), reactions with the electrolyte may get enhanced. Immersion of lithium metal anodes into various chemicals can allow engineering a desired surface composition, but the immersion process is time-consuming, *e.g.*, for ILs, an immersion time of 12 days is considered most favorable.^36^ Additionally, the native SEI influences the formation of the artificial SEI during immersion since the chemicals need to penetrate the native layer, which is also detrimental to homogeneity.^37^

**Figure 1:**
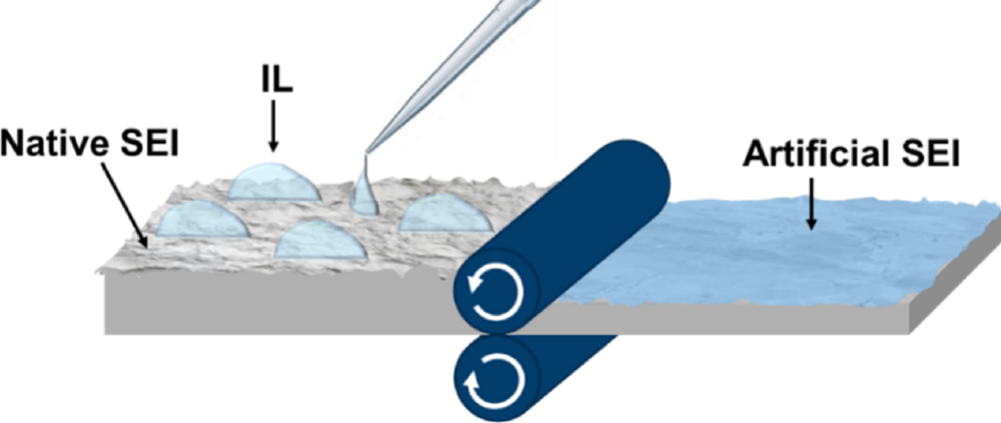
Schematic illustration of the mechanochemical lithium metal surface modification process.

Here, we propose a novel combined mechanical and chemical (= mechanochemical) approach that combines roll-pressing with immersion according to the process shown schematically in Figure 1. The lithium metal is roll-pressed in contact with a reactive solution. This method aims at enabling the chemicals to directly react with the “fresh” lithium surface created during roll-pressing and thereby avoiding reactions with oxygen or other constituents of the manufacturing environment (*i.e.*, dry air) and limiting the influence of the “native” SEI. Additionally, pressing further improves the contact between lithium metal and the immersion solution, which accelerates the reactions to form an artificial SEI. IL-based solutions are utilized since their nonvolatility is favorable for processing and they might induce beneficial properties to the SEI (such as the incorporation of cationic moieties that would act as an electrostatic shield or favorable conduction and mechanical properties).

To verify these hypotheses, lithium foils treated by the mechanochemical approach (Mechanochemical Modification) are compared with pristine foils (No Modification) or foils mechanically modified without IL (*i.e*., roll-pressed) followed or not by immersion in the IL for 1 or 7 days (called, respectively, Mechanical Modification, Mechanical + Immersion for 1 Day, and Mechanical + Immersion for 7 Days). All methods are applied prior to cell assembly with a liquid organic carbonate-based electrolyte to maintain high lithium mobility in the bulk, targeting high-voltage and low-temperature LMBs. As an immersion solution, *N*-butyl-*N*-methylpyrrolidinium bis(fluorosulfonyl)imide (Pyr_14_FSI) was chosen since this IL is already well studied and known to be beneficial for SEI formation on lithium metal.^38−40^ In this way, the beneficial effect of IL on the SEI to protect the lithium metal anode against further decomposition reactions and the high ionic conductivity of the carbonate-based electrolyte can be combined to enable superior room temperature performance.

Applying the mechanochemical modification led to significantly decreased impedance and low overvoltage during electrodeposition/-dissolution in symmetric Li∥Li cells, even at a high current density of 10 mA cm^–2^. Furthermore, the rate capability and the capacity retention in NMC∥Li cells could be significantly improved. Besides electrochemical investigations, X-ray photoelectron spectroscopy (XPS) was utilized directly after modification to shed light on the correlation between improved electrochemical performance and the composition of the artificial SEI layer, while scanning electron microscopy (SEM) was conducted after cycling to determine the morphology of the lithium metal surface. Furthermore, the morphology change during cycling was monitored by *operando* solid-state ^7^Li nuclear magnetic resonance (NMR) spectroscopy and the surface roughness of the lithium metal after applying different modification methods was compared by atomic force microscopy (AFM).

## 2 Results and Discussion

### 2.1 Electrochemical Performance in Symmetric Li∥Li Cells

**Figure 2:**
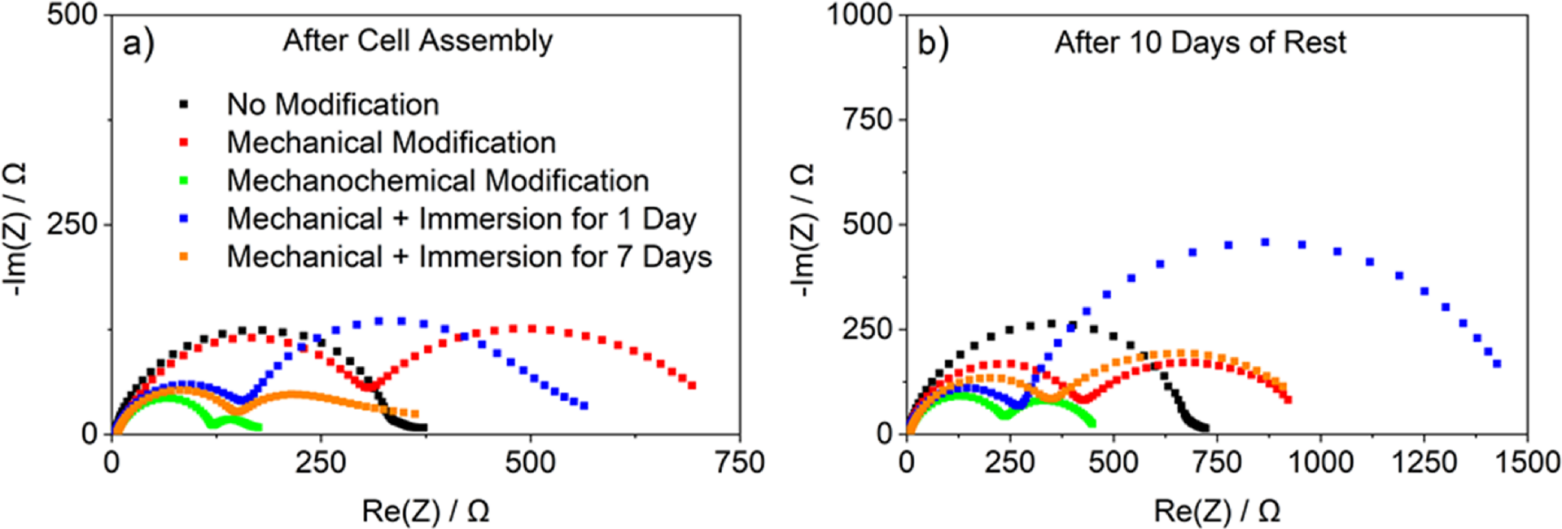
Nyquist plots of impedance spectra of symmetric Li∥Li cells with liquid carbonate-based electrolytes (1 M LiPF_6_ in EC:EMC (3:7)) (a) directly after cell assembly and (b) after 10 days under OCV conditions.

To evaluate the chemical stability of the modified surface layer against the organic carbonate-based electrolyte and its ability to passivate the lithium metal while still enabling sufficient ionic conductivity, symmetric Li∥Li cells were assembled and kept at open-circuit voltage (OCV) while the evolution of the SEI resistance was monitored by electrochemical impedance spectroscopy (EIS) for 10 days. The Nyquist plots of cells assembled with lithium metal foils subjected to the different surface treatments measured directly after cell assembly and after 10 days of storage are shown in Figure 2. In both cases, the cell with the mechanochemically modified lithium shows the lowest impedance with 180 and 450 Ω, respectively. It is actually the only modification method that decreases the impedance compared to the pristine lithium (*i.e.*, 350 and 710 Ω). In the Nyquist plots of cells with pristine lithium, only one semicircle is visible while all other cells result in two semicircles. A possible reason might be an additional contribution to the interphase resistance, *e.g.*, due to reactions with the Mylar foil during roll-pressing. At first, the mechanically modified lithium exhibits the largest impedance (700 Ω), which is governed by a lower increase during rest (920 Ω after 10 days). This can be explained by a “thinner” native surface film on the lithium metal being less passivating and allowing a fast reaction with the electrolyte within seconds after cell assembly. Once those reactions resulted in an effective SEI, further electrolyte decomposition is basically suppressed, at least when the cell is not cycled. The cell with lithium electrodes immersed for 1 day after roll-pressing shows the most significant increase in impedance from 560 to 1420 Ω, which is mostly due to the growth of the second semicircle and indicates poor passivation. In contrast, a longer immersion time of 7 days leads to a lower starting impedance and to an increase from 360 to 910 Ω. The lower impedance after longer immersion time indicates that an immersion time of 1 day is insufficient for effective SEI formation. Reactions with the electrolyte are not suppressed, although they are much slower compared with the mechanically modified lithium electrodes, and the contact with electrolyte leads to a significant increase in impedance. This observation is in accordance with the literature that suggests an even longer immersion time of 12 days.^36^

**Figure 3:**
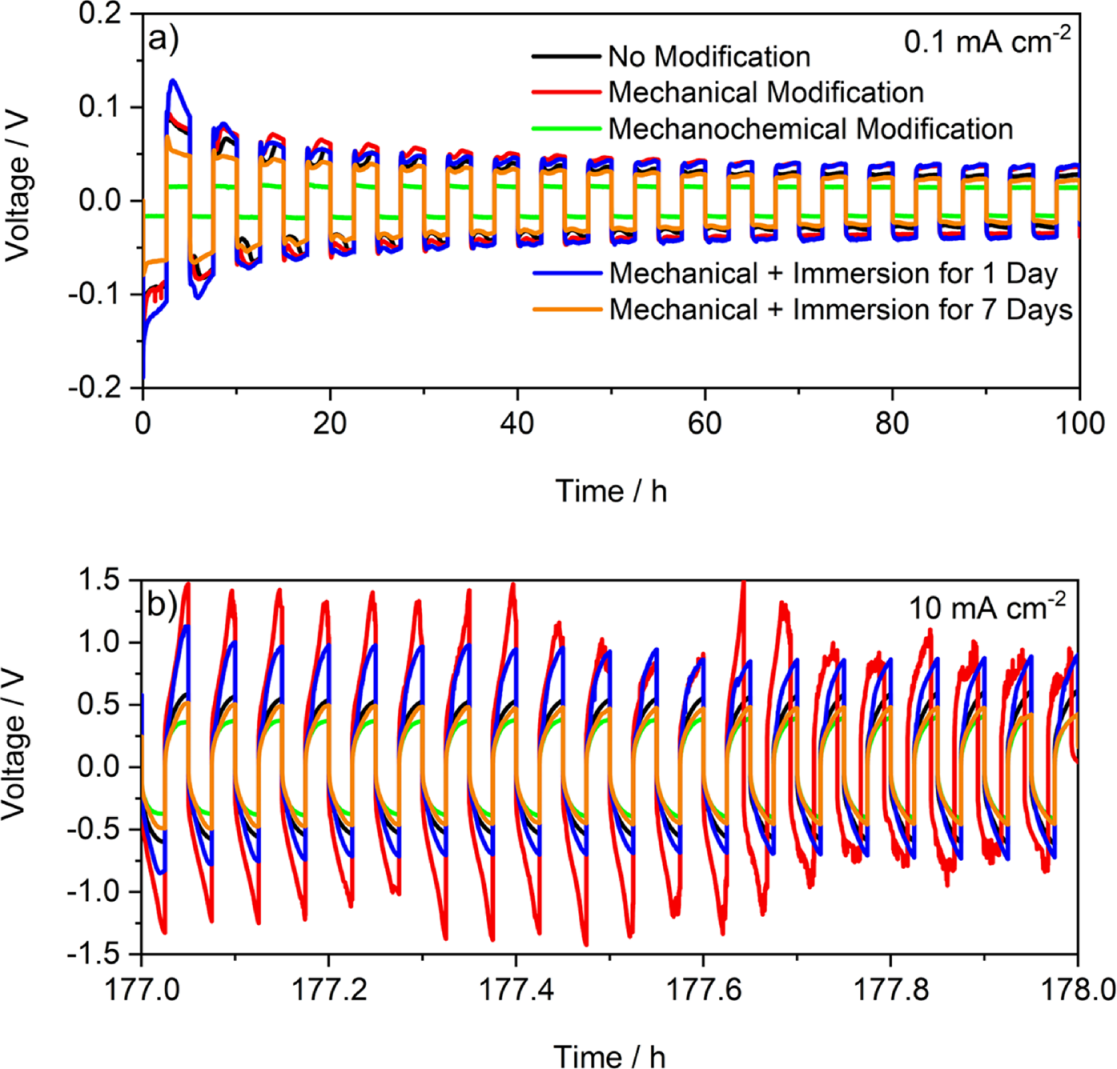
Overvoltage evolution of symmetric Li∥Li cells with liquid carbonate-based electrolytes (1 M LiPF_6_ in EC:EMC (3:7)) (a) at a current density of 0.1 mA cm^–2^ and (b) at 10 mA cm^–2^.

After confirming that the mechanochemical modification indeed lowers the impedance and its increase at OCV and therefore seems to be effective in limiting unwanted side reactions, the performance of the different treatments was further investigated in symmetric Li∥Li cells. The overvoltage evolutions at 0.1 and 10 mA cm^–2^ are presented in Figure 3. Voltage profiles at additional current densities can be found in Figure S2. Usually, symmetric Li∥Li cells exhibit relatively large overvoltages at the beginning of the first cycle due to the passivation layer at the electrode where electrodeposition occurs. Afterward, the overvoltage decreases as the surface is changed by the first deposition (*i.e.*, the SEI properties are modified by the passage of lithium, by stretching or cracking, exposing “fresh” lithium to the electrolyte). Those inhomogeneous changes lead to inhomogeneous electrodeposition/-dissolution, causing HSAL formation. During ongoing cycling, the surface area is further increased and therefore the actual areal current density is decreased, and hence the overvoltage decreases.^8^ Here, the cells with pristine, mechanically modified, and immersed lithium all follow this trend (Figure 3a) with obvious irregularities in each step (the cell internal resistance varies as less resistive HSAL grows or is disconnected from the electrodes). Remarkably, the cell with mechanochemically modified lithium exhibits a very low overvoltage of 0.015 V from the beginning with no change during cycling and stable voltage within each step. This strongly suggests that the SEI allows fast and homogeneous lithium ion transport, which leads to homogeneous electrodeposition/-dissolution and to the suppression of HSAL formation. Even at a high current density of 10 mA cm^–2^, the mechanochemically modified lithium shows a very smooth voltage profile with an overvoltage below 0.4 V that slightly increases within each step (in agreement with the formation of a concentration gradient in the bulk electrolyte) but does not increase from the first to the 20th cycle (Figure 3b). In contrast, the mechanically modified lithium reaches a cutoff voltage of 1.5 V and afterward displays a noisier voltage profile, indicating electrolyte consumption or even micro short circuits in the cell. The overvoltage of the cell with pristine lithium is ∼0.6 V and does not change significantly. The two cells with immersed lithium exhibit decreasing overvoltage during cycling, indicating HSAL formation. Noticeably, the cells with lithium immersed for 7 days have a lower overvoltage (∼0.5 V) than those immersed for 1 day (∼1.0 V), further supporting that an immersion time of 1 day is insufficient.

In comparison to pristine lithium metal anodes and other treatment methods, the relatively fast mechanochemical modification is by far the most effective in forming an artificial SEI that allows significantly faster and more homogeneous lithium deposition and thus provides improved interfaces and cycling performance due to effective SEI formation.

### 2.2 Surface Characterization

#### 2.2.1 Surface Composition

To get further insights into the origin of the improved SEI properties, the composition of the surface layer directly after pretreatment was determined by XPS. Several studies have already reported on the possible decomposition reactions of Pyr_14_FSI with lithium metal, where the anion is the main participant in SEI formation by decomposing into LiF and SO_2_NSO_2_F. The latter one leads in several reaction steps to the formation of Li*_x_*S*_y_*O*_z_* species.^28,34,38,41^ Moreover, the cation, although being chemically more stable than, *e.g.*, imidazolium cations, can also be reduced and likely participates in the formation of polymeric organic species in the SEI via the production of unsaturated and radical species formed by Hoffman β-elimination or 2-electron reduction. Hence, the inorganic SEI layer formed by anion decomposition is covered by an organic layer partly originating from the decomposition of the cation.^42^

**Figure 4:**
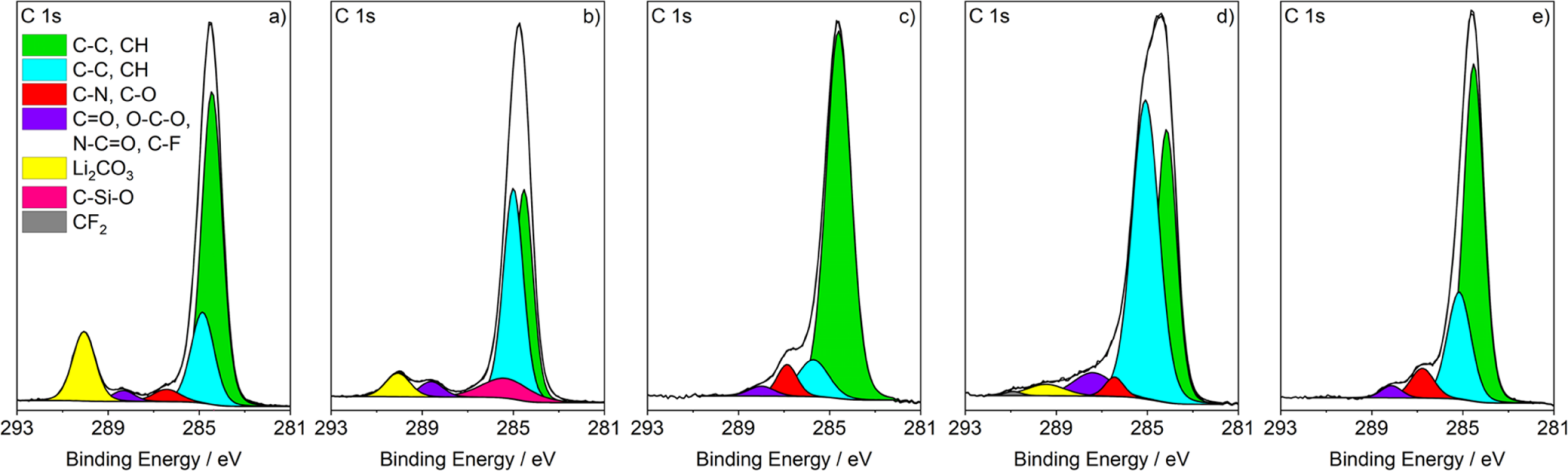
C 1s XPS spectra of lithium electrodes (a) without modification, (b) after mechanical modification, (c) after mechanochemical modification, and (d) after roll-pressing and immersion for 1 day and (e) for 7 days.

**Figure 5:**
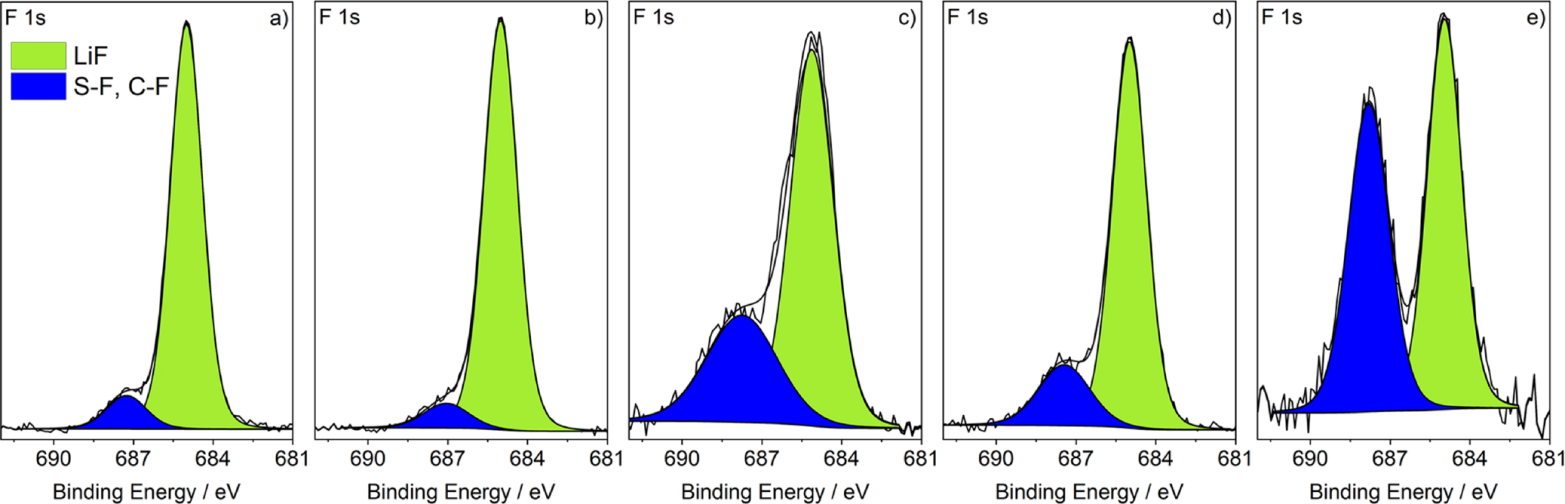
F 1s XPS spectra of lithium electrodes (a) without modification, (b) after mechanical modification, (c) after mechanochemical modification, and (d) after roll-pressing and immersion for 1 day and (e) for 7 days.

The C 1s and F 1s spectra are shown in Figures 4 and 5. In addition to the usual C–C/C–H peaks between 284 and 285 eV,^31,36^ all C 1s spectra display two smaller peaks at 286.5 and 288 eV, which can be ascribed to C–O/C–N bonds and other bonds to heteroatoms, respectively.^31,36^ The C 1s spectrum of pristine lithium exhibits a large peak at a binding energy of 290.0 eV, which can be assigned to Li_2_CO_3_ from the native SEI (Figure 4a).^31,36^ Mechanical modification leads to a decreased intensity of the Li_2_CO_3_ peak; however, a new peak at 285.3 eV arises, which might be attributed to polysiloxanes from the Mylar foil (Figure 4b). The C 1s spectra of the mechanochemically modified lithium and the lithium that was immersed for 7 days look similar. Both have an increased intensity of the C–O/C–N peak compared to pristine lithium, indicating that the cation of the IL or its decomposition products contribute to the surface layer. No Li_2_CO_3_ can be detected, whereas the spectrum of lithium that was only immersed for 1 day still exhibits a small peak at 290.0 eV. Additionally, traces of CF_2_ are detected at 291 eV.^43^ Furthermore, for this modification, the peak ratio between C–O/C–N bonds and other heteroatom bonds is inversed compared to the other modifications. The low intensity of the peak at 286.5 eV and the presence of Li_2_CO_3_ confirm that an immersion time of 1 day is insufficient to incorporate significant amounts of the IL cation (or its decomposition products) into the surface layer.

The F 1s spectra of pristine and mechanically modified lithium (Figure 5a,b) show one major peak at 685.0 eV assigned to LiF,^31,36^ which likely originates from the production process of lithium metal foil, while there are only traces of other fluorine species (687.5 eV^44^). After an immersion time of 1 day, the intensity of the peak at 687.5 eV is increased, and after 7 days of immersion, the peak has a similar intensity to the LiF peak. This peak can be attributed to the S–F bond of the undecomposed FSI anion, suggesting that, after the initial reaction between lithium metal and the anion during which LiF is formed, the surface is passivated and the anion is incorporated into the outer layer of the SEI. This process is more pronounced after 7 days of immersion; however, also the mechanochemically modified lithium exhibits a significant intensity for the S–F peak.

The species detected with XPS in this work are in general in accordance with the decomposition products reported in the literature. Considering the atomic ratios between the various elements (Table S1), increased nitrogen and silicon and decreased oxygen concentrations seem to be beneficial for an effective SEI, whereas the amount of fluorine species appears to have a minor influence. However, the ratio between the different fluorine species occurs to be of importance, a higher ratio of non-LiF fluorine species seems favorable. This is in contrast with several recent reports in the literature claiming that a LiF-rich SEI is the key to homogeneous electrodeposition/-dissolution and therefore improved electrochemical performance.^45−47^ LiF is known to be a major component of the inner SEI,^9^ but here, due to the low detection depth of XPS, we mainly investigated the outer layer of the SEI. Thus, it seems that the composition of the outer SEI and its changes after various lithium metal modification procedures are indicative of SEI performance.

#### 2.2.2 Surface Roughness

**Figure 6:**
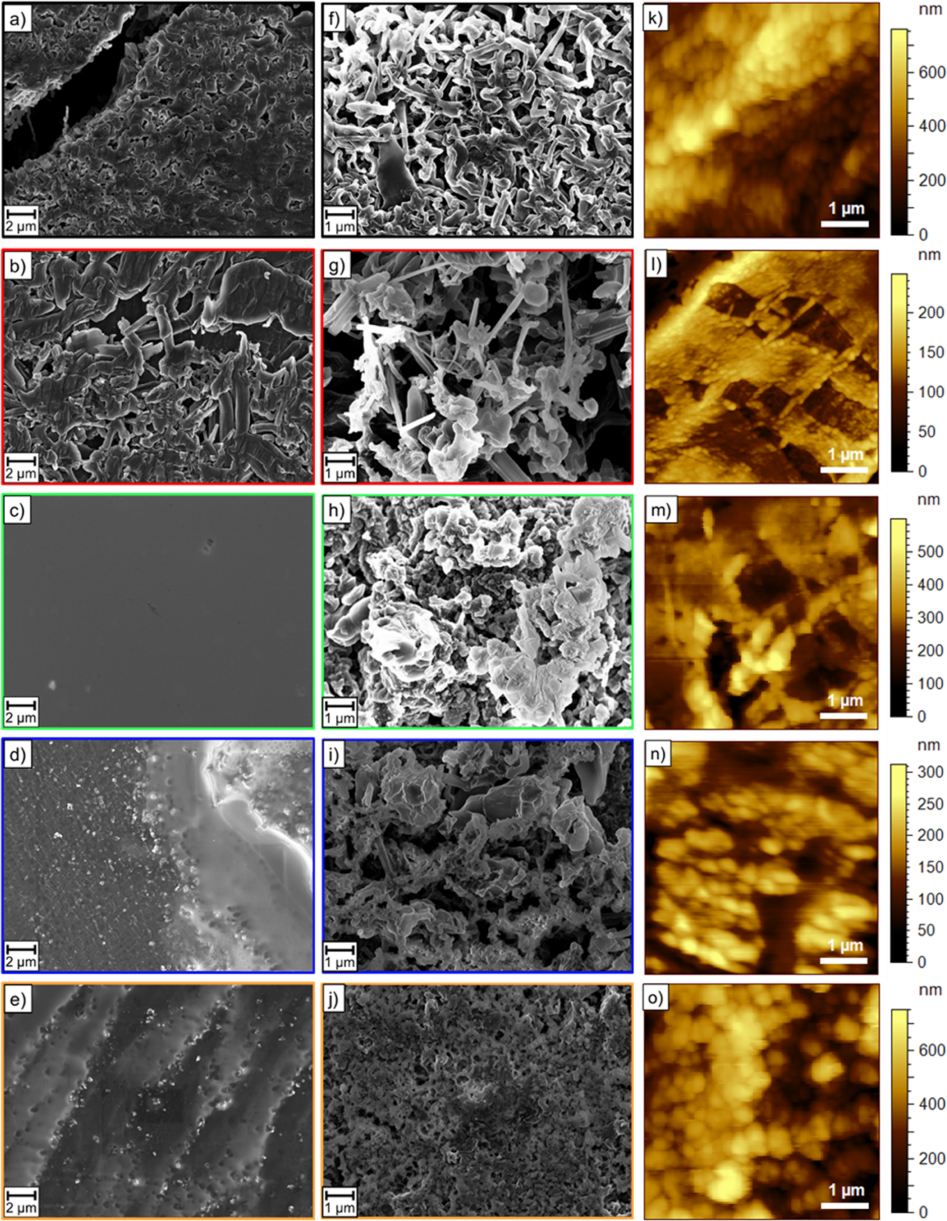
SEM images of lithium electrodes after cycling in symmetric Li∥Li cells with liquid carbonate-based electrolytes (1 M LiPF_6_ in EC:EMC (3:7)) (a, f) without modification, (b, g) after mechanical modification, (c, h) after mechanochemical modification, and (d, i) after roll-pressing and immersion for 1 day and (e, j) for 7 days after (a–e) one electrodeposition step (1 mAh, 1 mA cm^–2^) and (f–j) after 50 cycles (1 mAh, 1 mA cm^–2^). AFM topography images of (k) pristine lithium, (l) mechanically modified lithium, (m) mechanochemically modified lithium, and (n) lithium immersed for 1 day and (o) for 7 days.

In order to reveal differences in terms of surface roughness, AFM measurements were conducted directly after lithium surface modification. The topography images are shown in Figure 6, and the average surface roughness as well as the maximal surface roughness can be seen in Table S2. Pristine lithium exhibits an average surface roughness of 137 nm and a maximal surface roughness of 1090 nm, whereas the average surface roughness can be significantly decreased by mechanical modification (24 nm). The topography image of the mechanically modified lithium reveals the existence of damages on the lithium surface (Figure 6l) where the surface has been desquamated due to the removal of the Mylar foil after modification, leading to a relatively high maximal surface roughness of 736 nm. Without IL acting as a lubricant, lithium sticks to the Mylar foil and is, in fact, difficult to remove. Improving this process would further decrease the average and maximal surface roughness. Mechanochemically modified lithium has an average surface roughness of 53 nm and therefore a higher average surface roughness than mechanically modified lithium due to the formation of the artificial SEI on the surface. However, the maximal surface roughness is decreased (599 nm) since the IL enables easier removing from the Mylar foil, and thus damages can be avoided. Lithium immersed for 7 days reveals a high average surface roughness of 110 nm and the lithium immersed for 1 day has an average surface roughness of 34 nm, suggesting that after the shorter immersion time, artificial SEI formation just started, whereas for the longer immersion time, a thick and inhomogeneous surface layer with a roughness close to that of the native surface film on the pristine lithium is formed. This is further confirmed by a large maximal surface roughness of 752 nm after 7 days of immersion. The lithium electrode immersed for 1 day exhibits the lowest maximal surface roughness (313 nm), suggesting that as mentioned above, artificial SEI formation just started, preferentially inside the fissures originating from the removal of the Mylar foil, leading to a filling of those fissures, which explains the lower maximal surface roughness. In contrast, artificial SEI formation on the mechanochemically modified lithium is further progressed, covering not only the fissures but the whole surface with a protective layer, resulting in a larger maximal roughness (599 nm) compared to the lithium immersed for 1 day. Therefore, mechanochemical modification seems to offer the best compromise between uniform coverage with the artificial SEI and surface roughness. A reason for this might also be the particle size of the SEI compounds. The mechanochemically modified lithium exhibits large particles and a smooth surface, whereas the immersed electrodes feature small round-shaped particles on the surface.

#### 2.2.3 Surface Morphology

After cycling, the lithium surface morphology was characterized by SEM to determine the influence of the SEI composition on the shape of the lithium deposits. In Figure 6, SEM images of lithium electrodes after a single electrodeposition step and after 50 cycles are shown (both with 1 mAh steps at 1 mA cm^–2^). After one electrodeposition step, the untreated lithium electrode exhibits a porous but rather dense deposit structure (Figure 6a), whereas the mechanically modified lithium has loose and partly needle-like deposits (Figure 6b). In contrast, on the mechanochemically modified lithium, no deposits can be observed because the surface is still covered with a smooth SEI film (Figure 6c), further confirming the results from the electrodeposition/-dissolution experiments that suggest a homogeneous and highly lithium ion-conductive SEI, preventing deposition on top of the SEI. Furthermore, the immersed electrodes show deposits that were growing through the SEI or accumulating on top of the SEI, indicating that the SEI formed by immersion is not as effective as the SEI formed by mechanochemical modification and therefore results in deposition on top of the SEI instead of enabling lithium ion transport through the SEI. However, the SEI on the lithium immersed for 7 days seems to be more homogeneous than the SEI on the lithium immersed for 1 day.

After 50 cycles, pristine lithium and mechanically modified lithium show needle-like lithium deposits although the pristine lithium still has a denser deposit morphology (Figure 6f,g). In contrast to those dendritic deposits, mechanochemically modified lithium and immersed lithium exhibit dense mossy deposits (Figure 6h–j), which are unlikely to penetrate through the separator, thus causing less safety issues. Nevertheless, there are some differences regarding the deposit size. Mechanochemically modified lithium reveals the largest deposits (several μm) due to the low overvoltage. The lower the overvoltage, the more selective is the deposition, leading to the growth of larger deposits, since the required energy for the growth of an existing deposit is lower than that for a new deposit formation.^48^ Moreover, larger deposits have a smaller surface area, thus leading to less reaction with the electrolyte. The lithium immersed for 7 days shows a rather smooth surface, but with small pores and thus *a priori* a larger surface area. Overall, the SEM images further confirm that mechanochemical modification has a beneficial influence on the lithium electrodeposition/-dissolution processes. It is likely that the homogeneity of the deposits could be improved further by a better controlled lithium metal processing (*i.e.*, rather than manual handling of foil and electrodes) since any defect can have a strong effect on the lithium deposition morphology.

**Figure 7:**
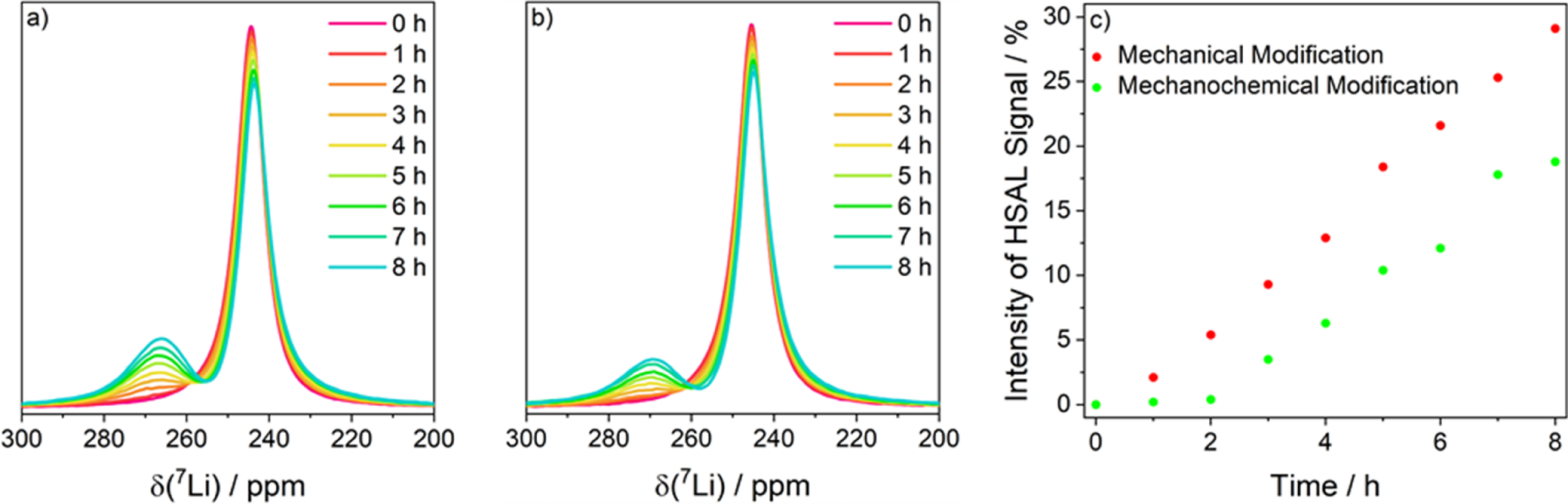
Metal region of the *operando* solid-state ^7^Li NMR spectra of symmetric Li∥Li pouchbag cells with liquid carbonate-based electrolytes (1 M LiPF_6_ in EC:EMC (3:7)) with (a) mechanically modified lithium and (b) mechanochemically modified lithium measured galvanostatically with an applied current density of 0.5 mA cm^–2^ for 8 h and (c) the time development of the relative HSAL signal intensity.

To further determine differences in the surface morphology, *operando* solid-state ^7^Li NMR was utilized. This method ensures that the morphology of the whole electrode is considered and not only selected areas as in SEM imaging. In Figure 7, the ^7^Li NMR spectra of mechanically modified lithium and mechanochemically modified lithium are shown. A constant current density of 0.5 mA cm^–2^ was applied to symmetric Li∥Li *operando* NMR pouchbag cells for 8 h, and ^7^Li NMR spectra were acquired every half hour. The signal of “bulk” lithium metal is located at a ^7^Li chemical shift of 245 ppm, whereas the signal at 265–270 ppm can be attributed to HSAL. For both modifications, the ^7^Li signal intensity for “bulk” lithium metal decreases over time while the signal intensity for HSAL increases (Figure 7a,b). The relative ^7^Li signal intensity of HSAL for the mechanically modified lithium increases almost linearly as a function of time and it reaches an intensity contribution of roughly 30% after 8 h, whereas the relative ^7^Li signal intensity of the HSAL for mechanochemically modified lithium increases significantly slower (Figure 7c). During the first 2 h, no observable formation of HSAL occurs. An approximately linear increase is visible over the following 5 h. However, in the last hour, there is only a very modest increase (note that the relative ^7^Li signal intensity of the HSAL is still below 20%). These results further confirm that the mechanochemical modification suppresses dendrite growth.

### 2.3 Electrochemical Performance in NMC∥Li Cells

**Figure 8:**
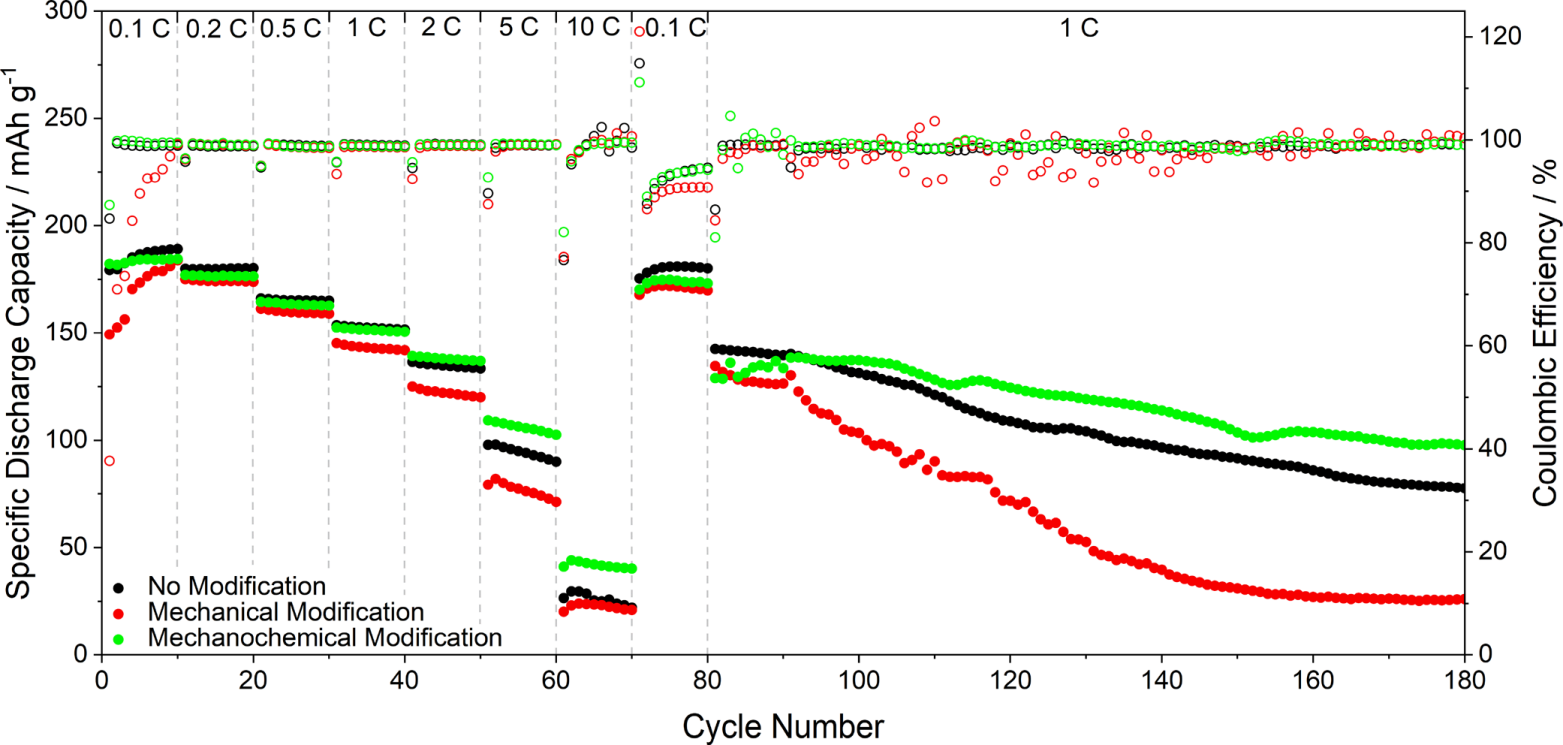
Specific discharge capacity (solid circles) and Coulomb efficiency (hollow circles) of NMC∥Li cells (with different lithium surface modifications) with liquid carbonate-based electrolytes (1 M LiPF_6_ in EC:EMC (3:7)) at various C-rates.

After observing an improved performance in symmetric Li∥Li cells and verifying those results by surface characterization that explains the reasons for the improvement, the mechanochemically modified lithium electrodes were cycled against LiNi_0.8_Mn_0.1_Co_0.1_O_2_ (NMC811) cathodes since a good compatibility with advanced cathode materials is crucial for application in industry. The specific discharge capacity and the CE of NMC∥Li cells tested at various C-rates are shown in Figure 8. The cell with mechanically modified lithium shows the poorest performance (25 mAh g^–1^ after 180 cycles). Especially during the first cycles, the specific discharge capacity (150 mAh g^–1^) and the CE (38%) are very low, most likely due to side reactions caused by insufficient SEI formation. The unmodified lithium shows slightly higher specific discharge capacities than the mechanochemically modified lithium at C-rates below 2 C, which might be explained by issues on the cathode side of the latter (*e.g.*, interference by soluble SEI components of the artificial SEI or some IL residues) or a favorable initial effect of HSAL formation with the unmodified lithium foil. However, the higher the C-rate and the longer the cycling, the more dendrites are formed. Thus, at 2 C or above, the advantage of dendrite suppression on the anode side prevails; hence, the mechanochemically modified lithium enables a higher specific discharge capacity (43 *vs* 27 mAh g^–1^ at 10 C). Furthermore, during ongoing cycling at 1 C after the C-rate test, the mechanochemically modified lithium exhibits less capacity decay (100 *vs* 80 mAh g^–1^ after 180 cycles).

Therefore, the cell with mechanochemically modified lithium not only shows the best rate capability but also the best capacity retention due to the suppression of dendrite growth and thus lower electrolyte consumption. To further improve the performance by tackling the issues on the cathode side, cathode electrolyte interphase (CEI) forming additives could be used.

## 3 Conclusions

In this work, a powerful method for the modification of lithium metal anodes was presented. It could be shown that the method influences not only the lithium metal surface composition and roughness after treatment but also the morphological changes during cycling and therefore the electrochemical behavior during electrodeposition/-dissolution. Remarkable differences in the chemical stability of the artificial SEI against the electrolyte are seen when comparing with roll-pressing followed by immersion in the same IL. The novel mechanochemical approach significantly improves the properties of the artificial SEI, leading to a stable cycling behavior, even at a high current density of 10 mA cm^–2^. Moreover, the impedance and therefore the interfacial resistance decreased and decomposition reactions with the organic carbonate-based electrolyte were suppressed, as shown by a lower impedance increase during OCV. The lithium ion conductivity of the artificial SEI formed by mechanochemical modification was significantly higher and more homogeneous than those by other modifications, enabling a more homogeneous electrodeposition/-dissolution and preventing dendrite growth through the SEI or the accumulation of deposits on top of the SEI as confirmed by SEM imaging. The mechanochemical modification method has a beneficial influence on the electrochemical behavior and is therefore a promising approach to overcome the drawbacks arising from the use of lithium metal anodes. Further improvements might be achieved by changing the IL or adding lithium salts to the solution or using other separators (since the lithium metal is pressed onto the separator micropores, which is detrimental for reaching fully homogeneous deposits) and, in general, improving any source of surface defect during the handling of the foils. Furthermore, this process enables easy upscaling, one of the main requirements for efficient application in industry. Finally, the mechanochemically modified lithium improves the rate capability as well as the capacity retention in NMC∥Li cells (the higher the C-rate, the more significant is the improvement compared to unmodified lithium), opening the door to commercialization of fast charging lithium metal batteries.

## 4 Experimental Section

### 4.1 Preparation of Modified Lithium Metal Anodes

For mechanical modification, the lithium metal foil (Albemarle, 500 μm) was rolled between two siliconized polyester foils (Mylar, PPI Adhesive Products Ltd., 100 μm) in 25 μm decrements using a tabletop roll-press (Hohsen Corp., HSAM-615H) until a thickness of 150 μm was reached. This process was carried out in a dryroom (dewpoints < −35 °C); afterward, the lithium was transferred into an argon-filled glovebox (H_2_O and O_2_ values <2 ppm) and electrodes (Ø 12 mm) were punched out. In the case of chemical modification, the electrodes were then immersed in *N*-butyl-*N*-methylpyrrolidinium bis(fluorosulfonyl)imide (Pyr_14_FSI, Solvionic, 99.9%, dried under vacuum at 80 °C for 3 days) for 1 or 7 days. For mechanochemical modification, the lithium metal foil was covered with Pyr_14_FSI (35 μL cm^–2^) prior to roll-pressing between Mylar foils.

### 4.2 Electrochemical Investigations

Symmetric Li∥Li coin cells^49^ (2032) were assembled in an argon-filled glovebox (H_2_O and O_2_ values <2 ppm) using a polypropylene-based microporous separator (Celgard 2500, Ø 16 mm, Celgard LLC, dried under vacuum at 40 °C for 2 days prior to assembly) wetted with 20 μL of LP57 electrolytes (1 M LiPF_6_ in EC (ethylene carbonate):EMC (ethyl methyl carbonate) 3:7, BASF) placed between two (modified or not) lithium disks (Ø 12 mm). NMC∥Li coin cells were assembled similarly, but the electrolyte content was increased to 50 μL to ensure sufficient wetting of the cathode and one lithium disk was replaced by a commercial LiNi_0.8_Mn_0.1_Co_0.1_O_2_ cathode (NMC811, Ø 12 mm, dried under vacuum for 24 h, 90% active material, 1 mAh cm^–2^, Custom Cells Itzehoe GmbH). All cells were measured at 20 °C, and several cells of each type have been investigated to ensure reproducibility. Symmetric Li∥Li cells cycling was carried out using a MACCOR battery test system (MACCOR Series 4000, MACCOR INC.) increasing the current density from 0.1 to 10 mA cm^–2^ (20 cycles per current density at 0.1, 0.25, 0.5, 1, 2, 5, and 10 mAh cm^–2^, then repeating the cycles at 0.1 and 1 mA cm^–2^) with a constant capacity of 0.25 mAh after an initial 12 h under OCV conditions to ensure sufficient wetting. For SEM characterization, the cells were cycled for one deposition step or 50 cycles at a current density of 1 mA cm^–2^ with a capacity of 1 mAh. Electrochemical impedance spectroscopy (EIS) was performed using a BioLogic VMP III potentiostat in a frequency range between 0.1 MHz and 0.1 Hz and an amplitude of 10 mV. The EIS measurements were started directly after cell assembly and continued under OCV conditions for 10 days. After an initial 12 h under OCV conditions to ensure sufficient wetting, NMC∥Li cells were cycled between 3.0 and 4.2 V using a MACCOR battery test system (MACCOR Series 4000, MACCOR INC.) increasing the C-rate from 0.1 to 10 C (10 cycles per C-rate) and then again 10 cycles at 0.1 C followed by 100 cycles at 1 C.

### 4.3 X-ray Photoelectron Spectroscopy

X-ray photoelectron spectroscopy (XPS) measurements were carried out at a 0° angle of emission and a pass energy of 20 eV using a monochromatic Al Kα source (*E*_photon_ = 1486.6 eV) with a 10 mA filament current and a filament voltage source of 12 kV. The analyzed area was approximately 300 μm × 700 μm. In order to compensate for the charging of the sample, a charge neutralizer was used. The F 1s peak at 685.0 eV was taken as an internal reference for the adjustment of the energy scale in the spectra. The fitting was carried out with CasaXPS. The samples were attached to the XPS sample holder with conductive carbon double-sided tape in an argon-filled glovebox directly after modification. Subsequently, the samples were transferred in sealed containers into a small glovebox attached to the XPS to avoid any contact to oxygen or moisture in the atmosphere before being placed in the ultravacuum chamber of the XPS. The instrument and the attached glovebox are operated using an ArW5 (Westfalengas, argon with 5% hydrogen).

### 4.4 Scanning Electron Microscopy

The surface morphology of the lithium metal anodes was characterized by scanning electron microscopy (SEM) using an Auriga field emission scanning electron microscope (FE-SEM) Crossbeam Workstation with a Schottky field emission gun (Carl Zeiss). The images were obtained with an in-lens secondary electron detector (In Lens SE) at an acceleration voltage of 3 kV and a working distance of about 3 mm. Prior to the measurements, the cells were disassembled in a glovebox with H_2_O and O_2_ values <2 ppm and dried under vacuum. The electrodes were then placed on sample holders with a sticky polymer conductive foil (Plano G3347) and transferred into the SEM device in an in-house-built air-tight sample holder to avoid any contact to oxygen and moisture in the atmosphere.

### 4.5 Nuclear Magnetic Resonance Spectroscopy

The morphology change during cycling was monitored by *operando* solid-state ^7^Li nuclear magnetic resonance (NMR) spectroscopy. These experiments were conducted with symmetric Li∥Li thin film pouch cells, which were assembled in a dryroom (dewpoints < −35 °C) adapted to a method described elsewhere.^50^ The lithium (mechanically modified or mechanochemically modified) was cut into electrodes (0.5 cm × 2.5 cm) and pressed onto copper mesh stripes, which were used as current collectors. Between the electrodes, a polypropylene-based separator (Celgard 2500, Celgard LLC, dried under vacuum at 40 °C for 2 days) wetted with 200 μL of LP57 electrolytes (1 M LiPF_6_ in EC (ethylene carbonate):EMC (ethyl methyl carbonate) 3:7, BASF) was placed. Coffee bag foils (Senseo) were used as cell casing, and the cells were hermetically sealed under vacuum. A constant current density of 0.5 mA cm^–2^ was applied for 8 h, and simultaneously, the ^7^Li NMR spectra were acquired every half hour. The ^7^Li NMR experiments were performed on a Bruker DSX spectrometer (Bruker) equipped with a widebore magnet operating at 200 MHz (4.7 T) using an in-house build broadband NMR probe, which allowed for electric operation of the cell inside the NMR magnet. The spectra were referenced to the ^7^Li resonance of an aqueous 1 M LiCl solution, which was set to 0 ppm. All experiments were performed at a resonance frequency of 77.8 MHz with a nutation frequency of 17.9 kHz. The recycle delay was set to 2 s. Spectral analyses and fitting of the data were performed using DmFit.

### 4.6 Atomic Force Microscopy

Atomic force microscopy (AFM) measurements were performed with a 5500 Atomic Force Microscope (Agilent Technologies) using an arrowshaped cantilever (PointProbe Plus ZEISS Veritekt Microscopes - Contact Mode Low Force Constant - Reflex Coating (PPP-ZEILR), Nanosensors, tip diameter < 10 nm). All images were recorded in the intermittent contact mode with constant force. The experiments were performed in a glovebox with argon flow to minimize contact to air. An area of 5 μm × 5 μm was chosen for all measurements. For data processing, the software MountainsSPIP (Digital Surf/Image Metrology) was utilized. The calculation of the arithmetic mean deviation of the surface roughness (called average surface roughness, *S*_a_, for simplicity) was done according to EUR 15178N. The maximal surface roughness (*S*_m_) is the difference between the highest and the lowest point on the sample surface within the region of interest.

## Supporting Information Available

Atomic ratios between the different elements on the lithium metal surface determined by XPS; average surface roughness and the maximal surface roughness measured by AFM; electrochemical data corresponding to the *operando* solid-state ^7^Li NMR spectra of symmetric Li∥Li thin film pouch cells; and voltage profiles of symmetric Li∥Li cells at additional current densities (PDF)
