# Supplementary material for: Effective Solid Electrolyte Interphase Formation on Lithium Metal Anodes by Mechanochemical Modification
Source: ACS Appl Mater Interfaces. 2021 Jul 15;13(29):34227–37. doi: 10.1021/acsami.1c07490 (PMC8397250; doi:10.1021/acsami.1c07490)
Supplement: Supplementary file 1 — am1c07490_si_001.pdf [file am1c07490_si_001.pdf]

Supporting Information

Effective SEI Formation on Lithium Metal Anodes by Mechanochemical Modification

Julia Wellmann,[†] Jan-Paul Brinkmann,[†] Björn Wankmiller,[‡] Kerstin Neuhaus,[†] Uta Rodehorst,[†] Michael R. Hansen,[‡] Martin Winter,[†,§] and Elie Paillard*[,†,◊]

[†]Forschungszentrum Jülich GmbH (IEK-12) Helmholtz-Institute Münster, Corrensstraße 46, 48149 Münster, Germany

[‡]Institute of Physical Chemistry, University of Münster, Corrensstraße 28-30, 48149 Münster, Germany

[§]MEET Battery Research Center, University of Münster, Corrensstraße 46, 48149 Münster, Germany

[◊]*Politecnico di Milano, Department of Energy, Via Lambruschini 4, 20156 Milan, Italy*

KEYWORDS. Ionic liquids, lithium metal anodes, lithium metal batteries, mechanochemical modification, solid electrolyte interphase

*Corresponding author: elieelisee.paillard@polimi.it

Table S1. Atomic ratios in the surface layer of lithium metal with different modifications determined by XPS.

	F (%)	O (%)	N (%)	C (%)	Li (%)	S (%)	Si (%)	P (%)
**No Modification**	4.6 (0.8)	19.5 (2.4)	0.00	32.9 (0.9)	42.6 (1.9)	0.00	0.00	0.30 (0.07)
**Mechanical Modification**	4.9 (0.6)	17.8 (1.2)	0.00	40.1 (4.0)	35.6 (2.5)	0.00	1.3 (0.4)	0.28 (0.06)
**Mechanochemical Modification**	3.5 (0.3)	13.5 (1.6)	2.3 (0.4)	49.9 (5.0)	21.7 (7.8)	2.7 (0.4)	6.3 (1.9)	0.00
**Mechanical + Immersed for 1 Day**	3.89	16.40	1.36	44.78	23.37	7.43	2.34	0.43
**Mechanical + Immersed for 7 Days**	3.4 (1.6)	10.7 (1.2)	2.6 (0.7)	57 (15)	22 (11)	3.3 (0.8)	0.26 (0.24)	0.00

Table S2. Arithmetic mean deviation of the surface roughness and maximal surface roughness after different modifications, calculated from the AFM topography images.

	Average Surface Roughness (S_a) (nm)	Maximal Surface Roughness (S_m) (nm)
**No Modification**	137	1090
**Mechanical Modification**	24	736
**Mechanochemical Modification**	53	599
**Mechanical + Immersed for 1 Day**	34	313
**Mechanical + Immersed for 7 Days**	110	752

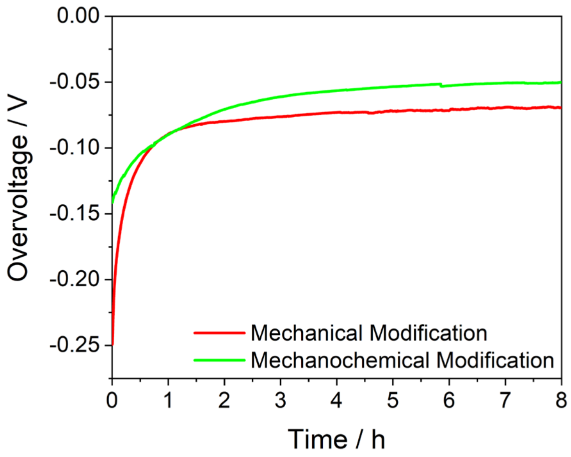

Figure S1. Overvoltage of symmetric Li‖Li pouchbag cells with liquid carbonate-based electrolyte (1 M $LiPF_6$ in EC: EMC (3:7)) at a current density of 0.5 mA cm^{-2} during *operando* ^{7}Li NMR measurements.

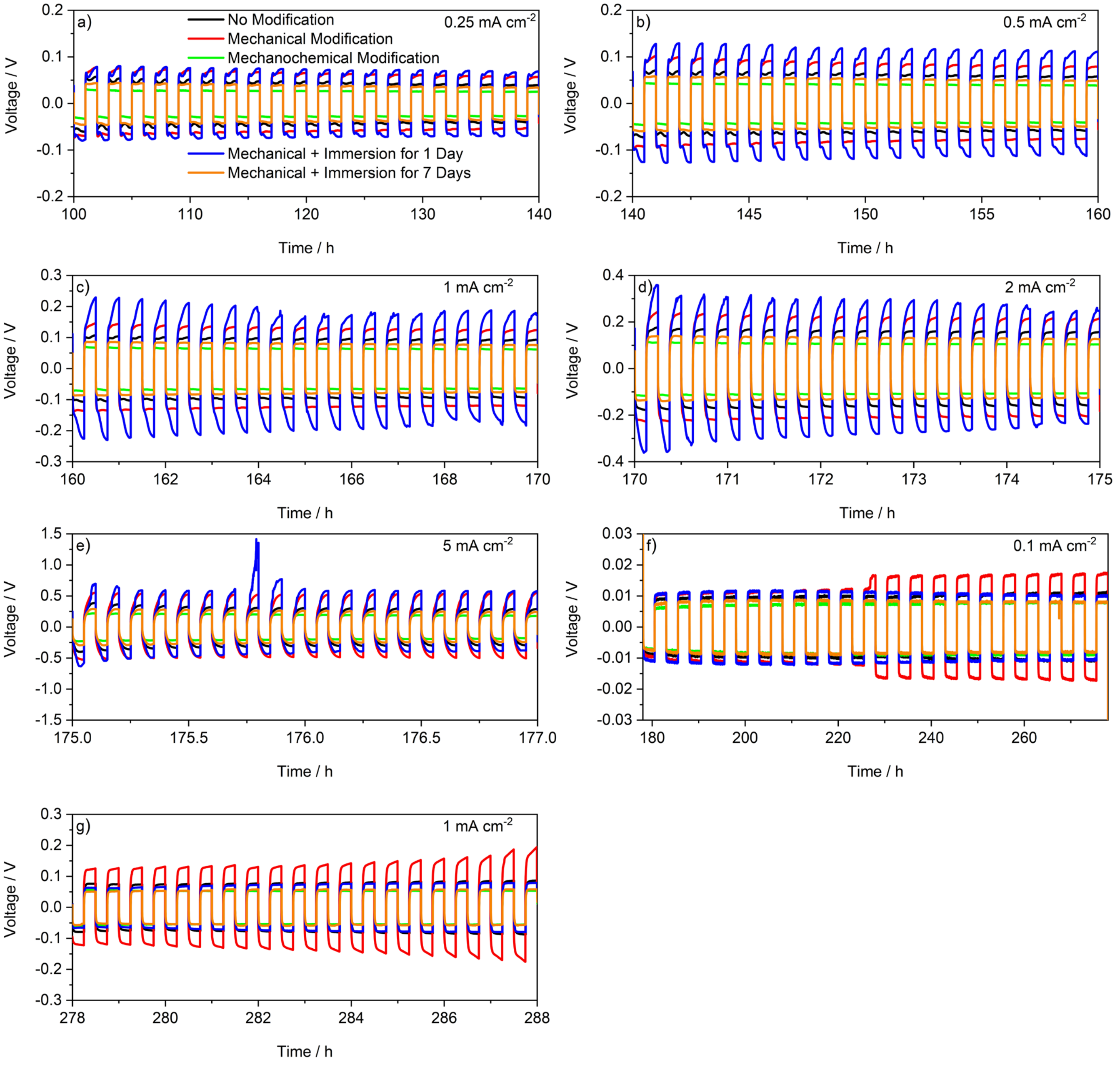

Figure S2: Overvoltage evolution of symmetric Li‖Li cells with liquid carbonate-based electrolyte (1 M LiPF6 in EC: EMC (3:7)) a) at a current density of 0.25 mA cm^{-2}, b) at 0.5 mA cm^{-2}, c) at 1 mA cm^{-2}, d) 2 mA cm^{-2}, e) 5 mA cm^{-2}, f) 0.1 mA cm^{-2}, and e) at 1 mA cm^{-2}.
